# Supplementary material for: Principal fitted component framework for robust support vector regression based on bounded loss: A simulation study with potential applications
Source: PLoS One. 2025 Jun 4;20(6):e0321102. doi: 10.1371/journal.pone.0321102 (PMC12142653; doi:10.1371/journal.pone.0321102)
Supplement: S1 File — S1 Table. The summary statistics (mean ± S.E) of MSE of regression coefficients regarding proposed and other studied estimators for p = 25 and ρ = 0.9. S2 Table. The summary statistics (mean ± S.E) of MAE of regression coefficients regarding proposed and other studied estimators for p = 25 and ρ = 0.9. S3 Table. The summary statistics (mean ± S.E) of MSE of regression coefficients regarding proposed and other studied estimators for p = 25 and ρ = 0.8. S4 Table. The summary statistics (mean ± S.E) of MAE of regression coefficients regarding proposed and other studied estimators for p = 25 and ρ = 0.8. S5 Table. The summary statistics (mean ± S.E) of MSE of regression coefficients regarding proposed and other studied estimators for p = 25 and ρ = 0.99. S6 Table. The summary statistics (mean ± S.E) of MAE of regression coefficients regarding proposed and other studied estimators for p = 25 and ρ = 0.99. S7 Table. A list of abbreviations used in the paper. (PDF) [file pone.0321102.s001.pdf]

S1 Table. The summary statistics (mean ± S.E) of MSE of regression coefficients regarding proposed and other studied estimators for *p*=25 and *ρ*=0.9.

	Sample size		Contamination levels		
Method	*n*	0%	5%	15%	30%
SVR	100<br>300<br>500	0.0785 ± 0.0245<br>0.0265 ± 0.0080<br>0.0157 ± 0.0048	0.0937 ± 0.0339<br>0.0280 ± 0.0086<br>0.0174 ± 0.0055	0.1399 ± 0.0491<br>0.0408 ± 0.0131<br>0.0227 ± 0.0060	0.5500 ± 0.3096<br>0.0949 ± 0.0374<br>0.0532 ± 0.0171
EQSVR	100<br>300<br>500	0.0013 ± 0.0003<br>0.0022 ± 0.0006<br>0.0021 ± 0.0006	0.0013 ± 0.0004<br>0.0020 ± 0.0006<br>0.0022 ± 0.0006	0.0015 ± 0.0004<br>0.0019 ± 0.0005<br>0.0025 ± 0.0006	0.0022 ± 0.0011<br>0.0018 ± 0.0005<br>0.0020 ± 0.0006
PCSVR	100<br>300<br>500	0.0053 ± 0.0034<br>0.0028 ± 0.0020<br>0.0020 ± 0.0012	0.0072 ± 0.0059<br>0.0031 ± 0.0022<br>0.0020 ± 0.0012	0.0103 ± 0.0075<br>0.0042 ± 0.0028<br>0.0025 ± 0.0018	0.0214 ± 0.0168<br>0.0089 ± 0.0061<br>0.0052 ± 0.0034
PCRSVR	100<br>300<br>500	0.0006 ± 0.0002<br>0.0004 ± 0.0002<br>0.0004 ± 0.0002	0.0007 ± 0.0003<br>0.0004 ± 0.0002<br>0.0004 ± 0.0002	0.0009 ± 0.0004<br>0.0004± 0.0002<br>0.0004 ± 0.0002	0.0019 ± 0.0012<br>0.0004± 0.0002<br>0.0004 ± 0.0003
PFCSVR	100<br>300<br>500	0.0011 ± 0.0016<br>0.0007 ± 0.0009<br>0.0004 ± 0.0005	0.0017 ± 0.0021<br>0.0008 ± 0.0011<br>0.0004 ± 0.0006	0.0025 ± 0.0036<br>0.0010 ± 0.0013<br>0.0006 ± 0.0009	0.0052 ± 0.0070<br>0.0021 ± 0.0030<br>0.0012 ± 0.0016
PFCRSVR	100<br>300<br>500	0.0004 ± 0.0002<br>0.0002 ± 0.0001<br>0.0001 ± 0.0001	0.0005 ± 0.0003<br>0.0001 ± 0.0001<br>0.0001 ± 0.0001	0.0007 ± 0.0003<br>0.0001 ± 0.0001<br>0.0001 ± 0.0001	0.0017 ± 0.0011<br>0.0001 ± 0.0001<br>0.0001 ± 0.0001

S2 Table. The summary statistics (mean ± S.E) of MAE of regression coefficients regarding proposed and other studied estimators for *p*=25 and *ρ*=0.9.

	Sample size		Contamination levels		
Method	*n*	0%	5%	15%	30%
SVR	100<br>300<br>500	0.2224 ± 0.0355<br>0.1295 ± 0.0202<br>0.0999 ± 0.0167	0.2442 ± 0.0448<br>0.1338 ± 0.0226<br>0.1066 ± 0.0175	0.2996 ± 0.0550<br>0.1591 ± 0.0255<br>0.1207 ± 0.0164	0.5759 ± 0.1592<br>0.2434 ± 0.0481<br>0.1836 ± 0.0307
EQSVR	100<br>300<br>500	0.0296 ± 0.0041<br>0.0375 ± 0.0059<br>0.0371 ± 0.0056	0.0299 ± 0.0051<br>0.0360 ± 0.0058<br>0.0383 ± 0.0061	0.0317 ± 0.0050<br>0.0352 ± 0.0051<br>0.0401 ± 0.0056	0.0405 ± 0.0114<br>0.0339 ± 0.0054<br>0.0361 ± 0.0061
PCSVR	100<br>300<br>500	0.0558 ± 0.0197<br>0.0399 ± 0.0152<br>0.0344 ± 0.0117	0.0638 ± 0.0265<br>0.0429 ± 0.0148<br>0.0347 ± 0.0116	0.0771 ± 0.0293<br>0.0493 ± 0.0183<br>0.0382 ± 0.0148	0.1098 ± 0.0435<br>0.0717 ± 0.0267<br>0.0553 ± 0.0197
PCRSVR	100<br>300<br>500	0.0213 ± 0.0052<br>0.0170 ± 0.0052<br>0.0157 ± 0.0052	0.0232 ± 0.0064<br>0.0165 ± 0.0049<br>0.0163 ± 0.0052	0.0261 ± 0.0060<br>0.0165 ± 0.0043<br>0.0171 ± 0.0053	0.0394 ± 0.0132<br>0.0168 ± 0.0046<br>0.0164 ± 0.0062
PFCSVR	100<br>300<br>500	0.0218 ± 0.0156<br>0.0173 ± 0.0127<br>0.0147 ± 0.0098	0.0286 ± 0.0189<br>0.0184 ± 0.0134<br>0.0142 ± 0.0098	0.0324 ± 0.0239<br>0.0215 ± 0.0154<br>0.0167 ± 0.0120	0.0477 ± 0.0345<br>0.0304 ± 0.0223<br>0.0236 ± 0.0165
PFCRSVR	100<br>300<br>500	0.0195 ± 0.0062<br>0.0112 ± 0.0044<br>0.0075 ± 0.0040	0.0216 ± 0.0071<br>0.0100 ± 0.0042<br>0.0079 ± 0.0047	0.0252 ± 0.0064<br>0.0102 ± 0.0040<br>0.0095 ± 0.0047	0.0392 ± 0.0133<br>0.0108 ± 0.0038<br>0.0098 ± 0.0052

S3 Table. The summary statistics (mean ± S.E) of MSE of regression coefficients regarding proposed and other studied estimators for p=25 and ρ=0.8.

	Sample size		Contamination levels		
Method	*n*	0%	5%	15%	30%
SVR	100	0.0445 ± 0.0135	0.0550 ± 0.0215	0.0875 ± 0.0311	0.5626 ± 0.4686
	300	0.0141 ± 0.0044	0.0152 ± 0.0047	0.0221 ± 0.0072	0.0530 ± 0.0214
	500	0.0084 ± 0.0025	0.0093 ± 0.0029	0.0121 ± 0.0031	0.0289 ± 0.0094
EQSVR	100	0.0020 ± 0.0005	0.0020 ± 0.0006	0.0022± 0.0005	0.0027 ± 0.0011
	300	0.0027 ± 0.0008	0.0025 ± 0.0008	0.0025 ± 0.0006	0.0024 ± 0.0007
	500	0.0023 ± 0.0006	0.0023 ± 0.0006	0.0028 ± 0.0007	0.0027 ± 0.0008
PCSVR	100	0.0032 ± 0.0021	0.0040 ± 0.0031	0.0058 ± 0.0041	0.0127 ± 0.0097
	300	0.0014 ± 0.0010	0.0017 ± 0.0011	0.0022 ± 0.0015	0.0048 ± 0.0032
	500	0.0010 ± 0.0007	0.0011 ± 0.0006	0.0013 ± 0.0009	0.0028 ± 0.0018
PCRSVR	100	0.0009 ± 0.0004	0.0010 ± 0.0005	0.0012 ± 0.0005	0.0020 ± 0.0012
	300	0.0005 ± 0.0003	0.0005 ± 0.0003	0.0005 ± 0.0002	0.0005 ± 0.0003
	500	0.0004 ± 0.0002	0.0004 ± 0.0003	0.0005 ± 0.0003	0.0005 ± 0.0002
PFCSVR	100	0.0007 ± 0.0010	0.0009 ± 0.0011	0.0013 ± 0.0019	0.0029 ± 0.0040
	300	0.0003 ± 0.0004	0.0004 ± 0.0005	0.0005 ± 0.0007	0.0012 ± 0.0016
	500	0.0002 ± 0.0002	0.0002 ± 0.0003	0.0003 ± 0.0005	0.0008 ± 0.0016
PFCRSVR	100	0.0006 ± 0.0003	0.0007 ± 0.0004	0.0009 ± 0.0004	0.0018 ± 0.0011
	300	0.00021± 0.0001	0.0002 ± 0.0004	0.0002 ± 0.0002	0.0002 ± 0.0001
	500	0.0001 ± 0.0001	0.0001 ± 0.0001	0.0001 ± 0.0001	0.0002 ± 0.0002

S4 Table. The summary statistics (mean ± S.E) of MAE of regression coefficients regarding proposed and other studied estimators for p=25 and ρ=0.8.

	Sample size		Contamination levels		
Method	*n*	0%	5%	15%	30%
SVR	100	0.1676 ± 0.0265	0.1867 ± 0.0356	0.2367 ± 0.0441	0.5645 ± 0.2111
	300	0.0947 ± 0.0154	0.0986 ± 0.0170	0.1172 ± 0.0188	0.1818 ± 0.0370
	500	0.0736 ± 0.0118	0.0779 ± 0.0127	0.0883 ± 0.0118	0.1353 ± 0.0229
EQSVR	100	0.0363 ± 0.0052	0.0364 ± 0.0058	0.0382 ± 0.0051	0.0436 ± 0.0104
	300	0.0416 ± 0.0068	0.0403 ± 0.0069	0.0398 ± 0.0060	0.0391 ± 0.0063
	500	0.0389 ± 0.0056	0.0389 ± 0.0063	0.0427 ± 0.0061	0.0414 ± 0.0066
PCSVR	100	0.0434 ± 0.0148	0.0475 ± 0.0192	0.0578 ± 0.0217	0.0846 ± 0.0331
	300	0.0286 ± 0.0105	0.0317 ± 0.0110	0.0363 ± 0.0133	0.0529 ± 0.0194
	500	0.0243 ± 0.0094	0.0256 ± 0.0084	0.0278 ± 0.0104	0.0410 ± 0.0142
PCRSVR	100	0.0260 ± 0.0059	0.0271 ± 0.0075	0.0306 ± 0.0066	0.0408 ± 0.0129
	300	0.0177 ± 0.0051	0.0182 ± 0.0053	0.0177 ± 0.0044	0.0189 ± 0.0056
	500	0.0157 ± 0.0056	0.0161 ± 0.0058	0.0179 ± 0.0059	0.0177 ± 0.0054
PFCSVR	100	0.0183 ± 0.0124	0.0209 ± 0.0133	0.0248 ± 0.0166	0.0355 ± 0.0260
	300	0.0126 ± 0.0189	0.0134 ± 0.0093	0.0158 ± 0.0114	0.0231 ± 0.0166
	500	0.0101 ± 0.0068	0.0106 ± 0.0069	0.0124 ± 0.0084	0.0178 ± 0.0117
PFCRSVR	100	0.0239 ± 0.0067	0.0252 ± 0.0082	0.0292 ± 0.0071	0.0402 ± 0.0128
	300	0.0113 ± 0.0050	0.0119 ± 0.0047	0.0118 ± 0.0040	0.0123 ± 0.0042
	500	0.0070 ± 0.0040	0.0084 ± 0.0044	0.0103 ± 0.0050	0.0107 ± 0.0064

S5 Table. The summary statistics (mean ± S.E) of MSE of regression coefficients regarding proposed and other studied estimators for *p*=25 and *ρ*=0.99.

	Sample size		Contamination levels		
Method	*n*	0%	5%	15%	30%
SVR	100	0.2582 ± 0.0746	0.2848 ± 0.0816	0.3377 ± 0.0931	0.5247±0.1444
	300	0.1726 ± 0.0530	0.1777 ± 0.0539	0.2334 ± 0.0676	0.4230 ±0.1427
	500	0.1198 ± 0.0341	0.1296 ± 0.0398	0.1641 ± 0.0430	0.3244 ±0.0990
EQSVR	100	0.0004 ± 0.0001	0.0004 ± 0.0002	0.0006± 0.0003	0.0022 ±0.0014
	300	0.0005 ± 0.0001	0.0004 ± 0.0001	0.0003 ± 0.0001	0.0003 ±0.0001
	500	0.0006 ± 0.0002	0.0006 ± 0.0004	0.0006 ± 0.0001	0.0005 ±0.0002
PCSVR	100	0.0288 ± 0.0186	0.0412 ± 0.0317	0.0526 ± 0.0378	0.0787 ±0.0531
	300	0.0207 ± 0.0150	0.0232 ± 0.0165	0.0306 ± 0.0196	0.0539 ±0.0343
	500	0.0159 ± 0.0096	0.0161 ± 0.0103	0.0205 ± 0.0154	0.0377 ±0.0253
PCRSVR	100	0.0003 ± 0.0002	0.0004 ± 0.0002	0.0006 ± 0.0003	0.0020 ±0.0014
	300	0.0001 ± 0.00008	0.0001 ± 0.00008	0.0001 ± 0.00007	0.0001±0.00007
	500	0.0001 ± 0.00009	0.0001 ± 0.0001	0.0001 ± 0.0001	0.0001 ± 0.0001
PFCSVR	100	0.0064 ± 0.0106	0.0094 ± 0.0120	0.0130 ± 0.0192	0.0243 ± 0.0332
	300	0.0053 ± 0.0067	0.0059 ± 0.0096	0.0077 ± 0.0106	0.0135 ± 0.0190
	500	0.0039 ± 0.0049	0.0038 ± 0.0053	0.0049 ± 0.0079	0.0093 ± 0.0120
PFCRSVR	100	0.0003 ± 0.0001	0.0003 ± 0.0002	0.0005 ± 0.0003	0.0022 ± 0.0018
	300	0.00009±0.00006	0.00008± 0.0001	0.00008±0.00005	0.00009±0.00007
	500	0.00005±0.00004	0.00004±0.00004	0.00006±0.00005	0.0001 ± 0.0001

S6 Table. The summary statistics (mean ± S.E) of MAE of regression coefficients regarding proposed and other studied estimators for *p*=25 and *ρ*=0.99.

	Sample size		Contamination levels		
Method	*n*	0%	5%	15%	30%
SVR	100	0.4029 ± 0.0592	0.4284 ± 0.0655	0.4669 ± 0.0688	0.5801 ± 0.0864
	300	0.3304 ± 0.0526	0.3364 ± 0.0559	0.3813 ± 0.0577	0.5175 ± 0.0903
	500	0.2757 ± 0.0433	0.2903 ± 0.0462	0.3251 ± 0.0434	0.4509 ± 0.0705
EQSVR	100	0.0179 ± 0.0045	0.0190 ± 0.0054	0.0235 ± 0.0060	0.0447 ± 0.0147
	300	0.0181 ± 0.0027	0.0166 ± 0.0027	0.0157 ± 0.0023	0.0150 ± 0.0024
	500	0.0209 ± 0.0033	0.0203 ± 0.0059	0.0198 ± 0.0028	0.0177 ± 0.0045
PCSVR	100	0.1298 ± 0.0442	0.1529 ± 0.0622	0.1726 ± 0.0654	0.2144 ± 0.0757
	300	0.1085 ± 0.0407	0.1161 ± 0.0420	0.1322 ± 0.0468	0.1774± 0.0618
	500	0.0972 ± 0.0324	0.0967 ± 0.0337	0.1072 ± 0.0434	0.1479 ± 0.0539
PCRSVR	100	0.0172 ± 0.0055	0.0181 ± 0.0061	0.0229 ± 0.0061	0.0426 ± 0.0151
	300	0.0102 ± 0.0029	0.0099 ± 0.0027	0.0095 ± 0.0025	0.0099 ± 0.0027
	500	0.0097 ± 0.0030	0.0098 ± 0.0036	0.0097 ± 0.0033	0.0105 ± 0.0049
PFCSVR	100	0.0498 ± 0.0404	0.0641 ± 0.0458	0.0734 ± 0.0545	0.1012 ± 0.0761
	300	0.0465 ± 0.0363	0.0490 ± 0.0383	0.0557 ± 0.0428	0.0750 ± 0.0576
	500	0.0419 ± 0.0279	0.0401 ± 0.0294	0.0454 ± 0.0341	0.0625 ± 0.0458
PFCRSVR	100	0.0167 ± 0.0057	0.0184 ± 0.0060	0.0231 ± 0.0065	0.0435 ± 0.0173
	300	0.0080 ± 0.0030	0.0077 ± 0.0037	0.0077 ± 0.0030	0.0084 ± 0.0035
	500	0.0058 ± 0.0026	0.0054 ± 0.0025	0.0064 ± 0.0026	0.0078 ± 0.0058

S7 Table. A list of abbreviations used in the paper.

Full form	Abbreviation

ConCave-Convex Procedure	CCCP
Karush-Kuhn-Tucker	KKT
Exponential Quantile SVR	EQSVR
Mean Absolute Error	MAE
Mean Square Error	MSE
Mean of the Standard Errors of Bootstrap Estimates	MSEBE
Principal Components	PCs
Principal Component Analysis	PCA
Principal Component Regression	PCR
Principal Fitted Components	PFCs
Principal Fitted Component Regression	PFCR
Principal Component Robust Support Vector Regression	PCRSVR
Principal Fitted Component Robust Support Vector Regression	PFCRSVR
Standard Error	SE
Support Vector Machines	SVM
Support Vector Regression	SVR
Twin Support Vector Regression	TSVR
