# Supplementary figures and images for: Principal fitted component framework for robust support vector regression based on bounded loss: A simulation study with potential applications
Source: PLoS One. 2025 Jun 4;20(6):e0321102. doi: 10.1371/journal.pone.0321102 (PMC12142653; doi:10.1371/journal.pone.0321102)

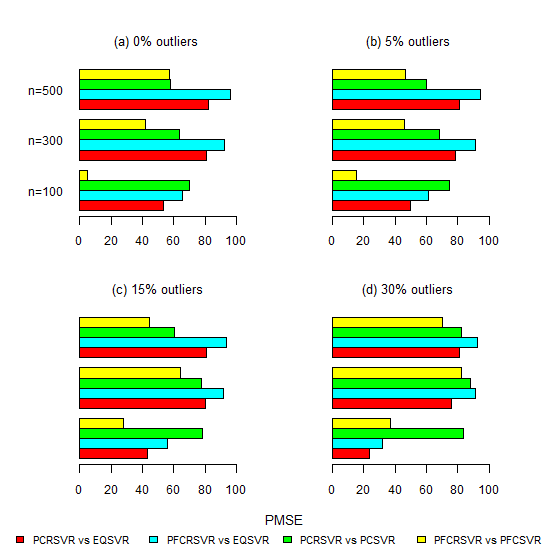

Supplement: S1 Fig — (TIFF) [file pone.0321102.s002.tiff]

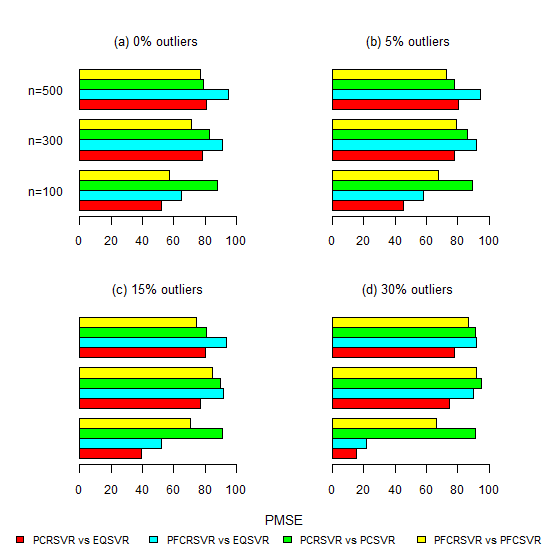

Supplement: S2 Fig — (TIFF) [file pone.0321102.s003.tiff]

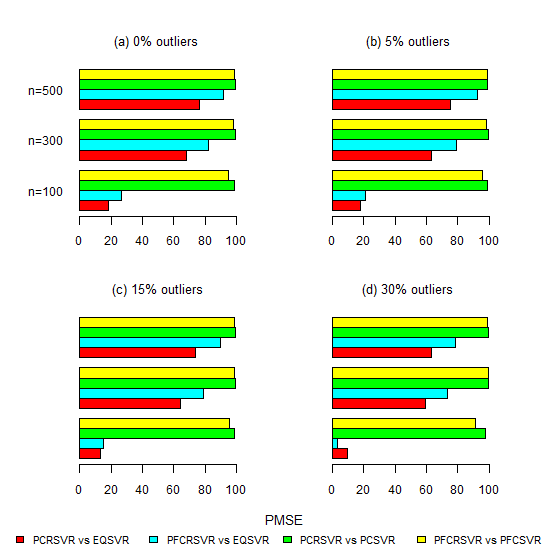

Supplement: S3 Fig — (TIFF) [file pone.0321102.s004.tiff]
