# Supplementary material for: Principal fitted component framework for robust support vector regression based on bounded loss: A simulation study with potential applications
Source: PLoS One. 2025 Jun 4;20(6):e0321102. doi: 10.1371/journal.pone.0321102 (PMC12142653; doi:10.1371/journal.pone.0321102)
Supplement: Attachment — Submitted filename: review comments PONE-D-24-60709_reviewer.pdf [file pone.0321102.s005.pdf]

TITLE: Principal fitted component framework for robust support vector regression based on bounded loss: A simulation study with potential applications

Manuscript Number: PONE-D-24-60709_reviewer

Type of manuscript: Original Article

Summary

In this paper, the authors present two modelling frameworks: Principal Component Robust Support Vector Regression (PCRSVR) and Principal Fitted Component Robust Support Vector Regression (PFCRSVR). These frameworks are expected to enhance estimation accuracy by integrating Exponential Quantile SVR with principal components and fitted components, effectively managing anomalies, ill-conditioned predictors, and high-dimensional data, as supported by simulation studies and real-life applications.

The novelty of the approach lies in how these elements are integrated to enhance robustness against anomalies and ill-conditioned predictors while maintaining effective performance in high-dimensional data settings. The specific focus on a "bounded loss" framework further indicates a unique contribution, as this may address issues of sensitivity in traditional SVR methods that can be adversely affected by outliers or noise. However, the manuscript requires some improvements. Some general comments are:

Comments

1. Arrange the keywords in alphabetic order.

2. Provide a list of abbreviations used in the paper.

3. At the end of all figure and table captions, insert a full stop.

4. List the study's highlights at the end of the "Introduction" section.

5. Software packages used should be cited to acknowledge the author(s).

6. I suggest you create a separate "Discussion" section. In the discussion section, the authors should present their findings and their main implications, highlight their study's limitations, and briefly mention some precise directions they intend to follow in their future research work. Can the authors mention how much of their research is influenced by the data they used or to which extent the methodology used within the developed research can be easily applied to other situations when the datasets differ? In this way, the authors could highlight the generalisation capability of their approach to justify a wider contribution to the current state of the art.

7. In the "Conclusion" section, authors should avoid summarising the aspects they have already stated in the body of the manuscript. Instead, they should interpret their findings at a higher level of abstraction than in the previous sections of the manuscript. The authors should highlight whether or to what extent they have addressed the necessity identified within the "Introduction" section (the identified gap). The authors should avoid restating everything they did once again. However, instead, they should emphasise what their findings mean to the readers, making the

"Conclusions" section interesting and memorable to them. The authors should not restate what they have done or what the article does. They should focus instead on what they have discovered and, most importantly, on what their findings mean.

8. The authors point out that a limitation of the study is the use of a narrow dataset range, which affects the generalizability of the findings. This could be expanded further to discuss how the specific characteristics of these datasets might skew results or what types of datasets could serve as valid tests for these frameworks.

9. The authors mention that the methods were tested only on continuous response variables assuming normality. This crucial point could have been elaborated on by discussing the implications of such limitations, such as the potential mismatch between method appropriateness and real-world data distribution.

10. While the focus on outliers in the response variable is commendable, the review could benefit from a deeper exploration of how leverage points in predictor space might impact the performance of these methods. This omission points to a significant blind spot in assessing the overall robustness of the proposed frameworks.

11. The assertion that the techniques are limited to scenarios where the number of observations exceeds the number of predictors is an important limitation that could be explored further. A brief discussion on the implications of applying these methods in high-dimensional settings could clarify the next research steps.

12. In the spirit of reproducible research, the authors should provide both the data and the codes. These can be deposited on GitHub, for example.
